# Supplementary material for: An Organic Fraction of Oenothera rosea L’Her Ex. Aiton Prevents Neuroinflammation in a Rat Ischemic Model
Source: Pharmaceuticals (Basel). 2024 Sep 9;17(9):1184. doi: 10.3390/ph17091184 (PMC11434707; doi:10.3390/ph17091184)
Supplement: Supplementary file 1 [file pharmaceuticals-17-01184-s001.zip › pharmaceuticals-3172899-supplementary.pdf]

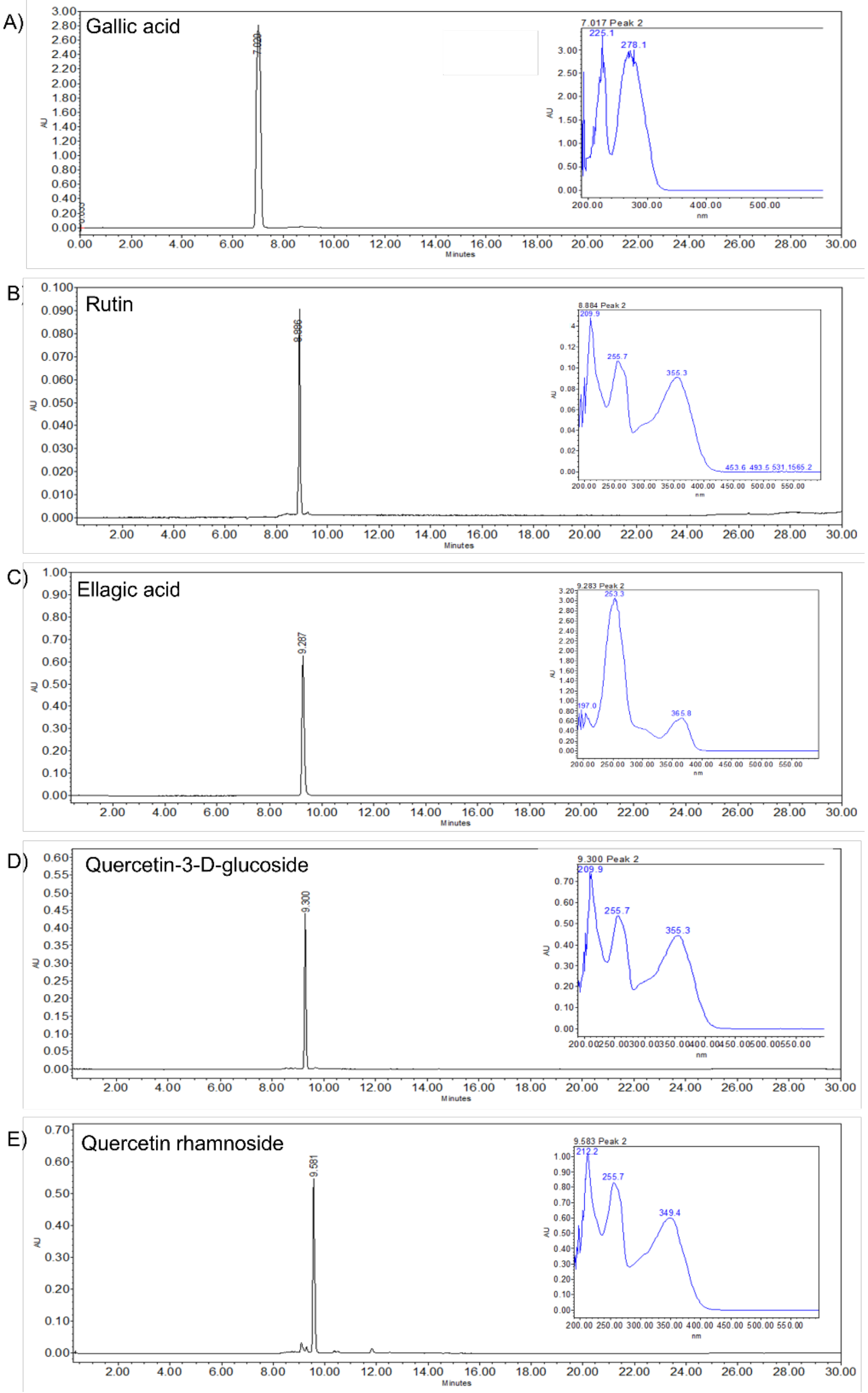

Figure S1. Commercial standards of chemical compounds present in *O. rosea*. HPLC chromatograms (detection at 300 *nm*)

Ellagic acid				
Concentration µg/mL	A1	A2	A3	Average
1.56	168198	167893	169469	168520
3.185	323500	322810	324645	323651.6667
6.25	614528	613402	614969	614299.6667
12.5	1167714	1167708	1166668	1167363.333
25	2249820	2247493	2250438	2249250.333

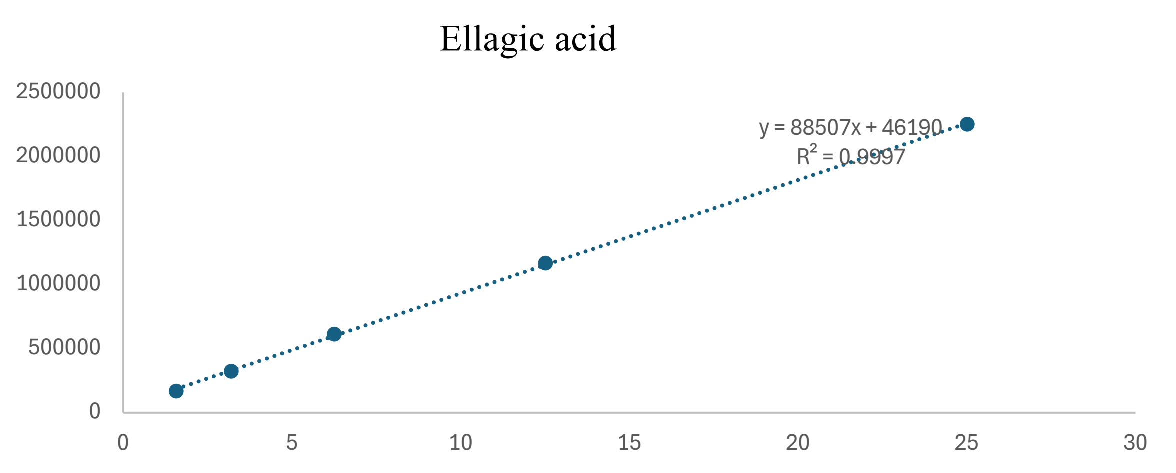

Sample	A1	A2	A3	Average	µg Ellagic acid /2 mg EAOr		mg Ellagic acid /g EAOr
EAOr	1067579	1066228	1058369	1064058.667	11.50043123	5750.215614	5.750215614

Gallic Acid				
Concentration µg/mL	A1	A2	A3	Average
12.5	158093	158012	158420	158175
25	290494	289594	289599	289895.6667
50	598847	599390	599516	599251
100	1190895	1191397	1191397	1191229.667
200	2392174	2394127	2388810	2391703.667

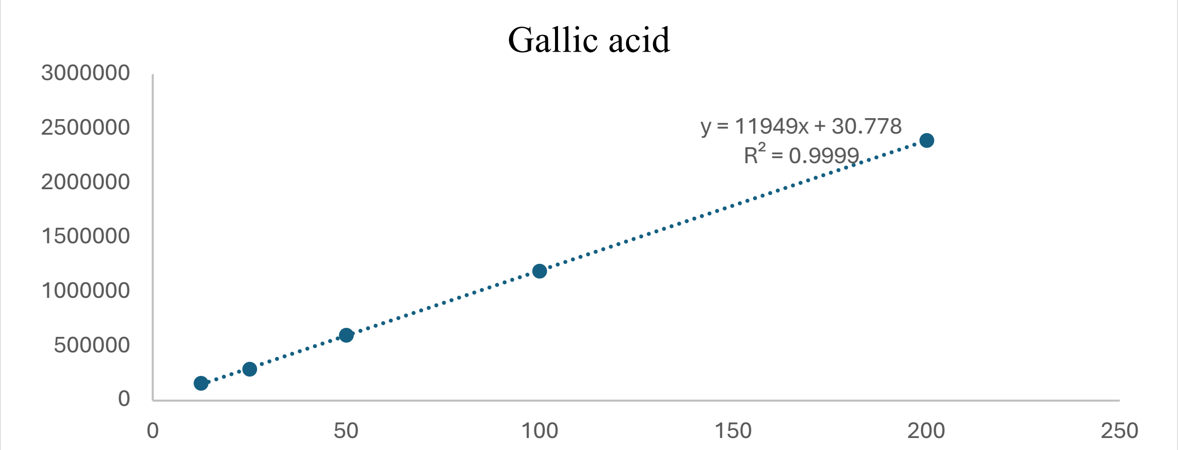

Quantitative analysis							
Sample	A1	A2	A3	Average	µg Gallic acid /2 mg EAOr		mg Gallic acid / g EAOr
EAOr	286794	287496	284963	286417.6667	21.394231	10697.1155	10.6971155

**Kaempferol**				
Concentration µg/mL	A1	A2	A3	Average
25	667130	668853	665735	667239.3333
50	1334887	1341985	1338806	1338559.333
100	2609272	2573074	2577979	2586775
200	5181000	5179225	5233357	5197860.667

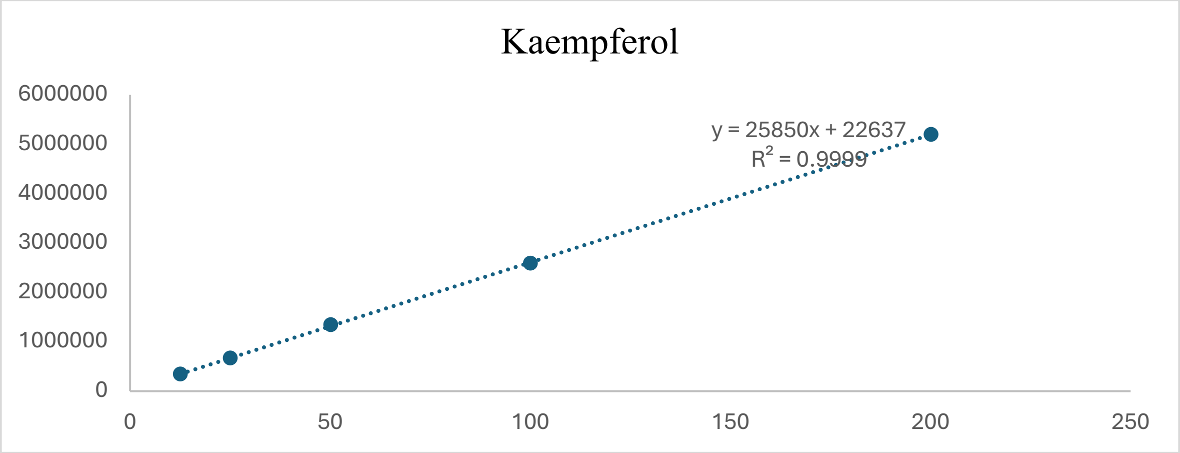

Quantitative analysis							
Sample	A1	A2	A3	Average	µg kaempferol /2 mg EAOr		mg Kaempferol/(g EAOr
EAOr	65698	60785	59017	61833.33333	1.516299162	758.1495809	0.758149581

Quercetin				
Concentration µg/mL	A1	A2	A3	Average
12.5	310432	310001	309728	310053.6667
25	632981	632981	629640	631867.3333
50	1269761	1272110	1269761	1270544
100	2556397	2556635	2554611	2555881
200	5334884	5339523	5334884	5336430.333

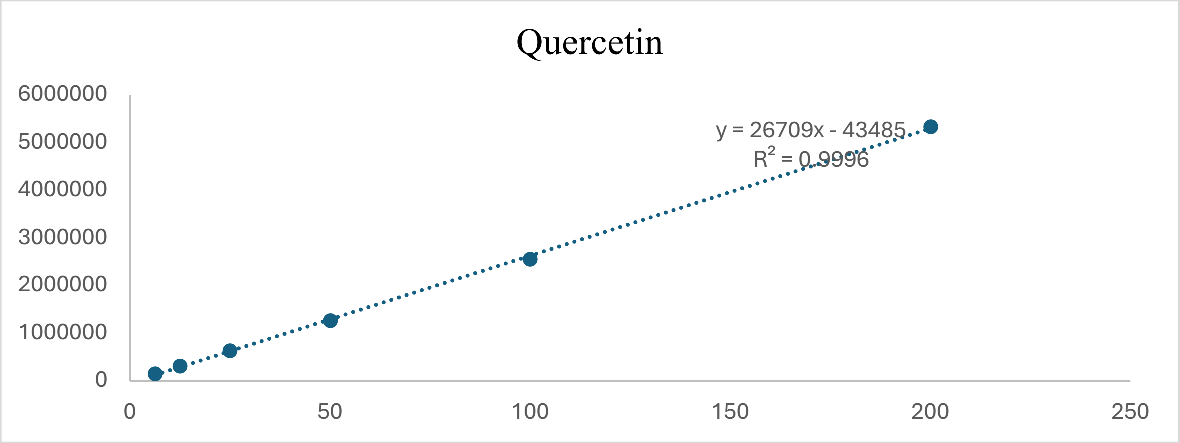

Quantitative analysis							
Sample	A1	A2	A3	Average	µg Quercetin/ 2 mg EAOr		mg Quercetin/ g EAOr
EAOr	152785	152813	146666	150754.6667	7.2724425	3636.22125	3.63622125

Quercetin Rhamnoside				
Concentration µg/mL	A1	A2	A3	Average
12.5	27557	27557	27557	27557
25	47413	88395	89271	75026.33333
50	210507	209469	210835	210270.3333
100	496778	497248	497248	497091.3333
200	905601	914895	914630	911708.6667

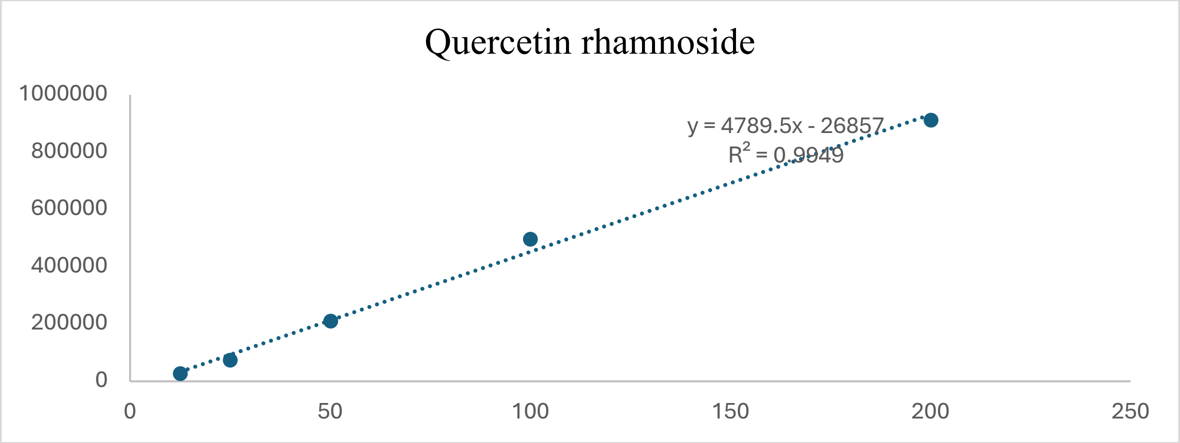

Quantitative analysis							
Sample	A1	A2	A3	Average	µg Quer. Rham/2 mg EAOr		mg Quercetin Rham/g EAOr
EAOr	116223	117891	119288	117800.6667	30.20308313	15101.54157	15.10154157

Quercetin glucoside				
Concentration µg/mL	A1	A2	A3	Average
12.5	217591	214539	217591	216573.6667
25	442984	442269	442269	442507.3333
50	891697	891697	899183	894192.3333
100	1773753	1779211	1786301	1779755
200	3463939	3485631	3440837	3463469

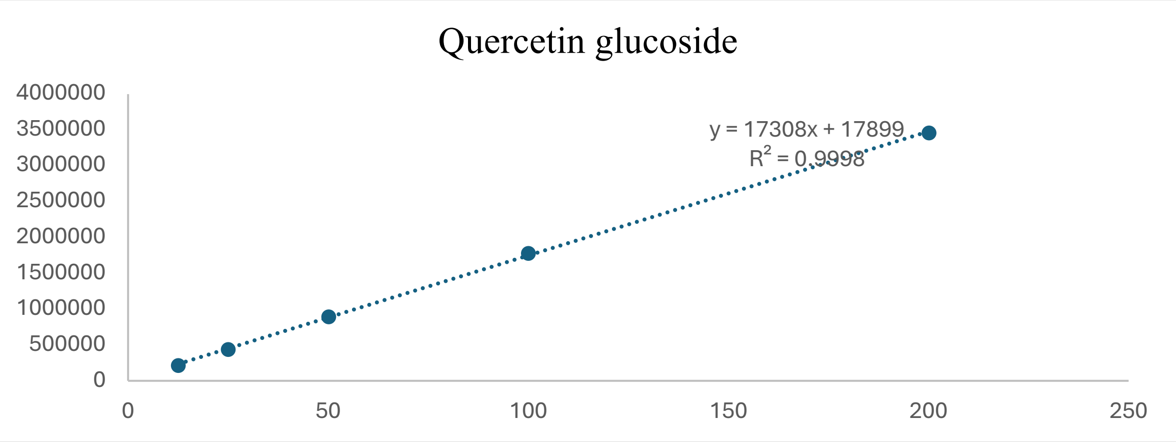

Quantitative analysis							
Sample	A1	A2	A3	Average	µg Quercetin Glu/2 mg EAOr		mg Quercetin Glu/g EAOr
EAOr	58773	57801	58773	58449	2.34284724	1171.42362	1.17142362

Rutin				
Concentration µg/mL	A1	A2	A3	Average
12.5	322897	318605	318605	320035.6667
25	633399	633399	599930	622242.6667
50	1203410	1203410	1205410	1204076.667
100	2423828	2185067	2521604	2376833
200	4906466	4921065	4875868	4901133

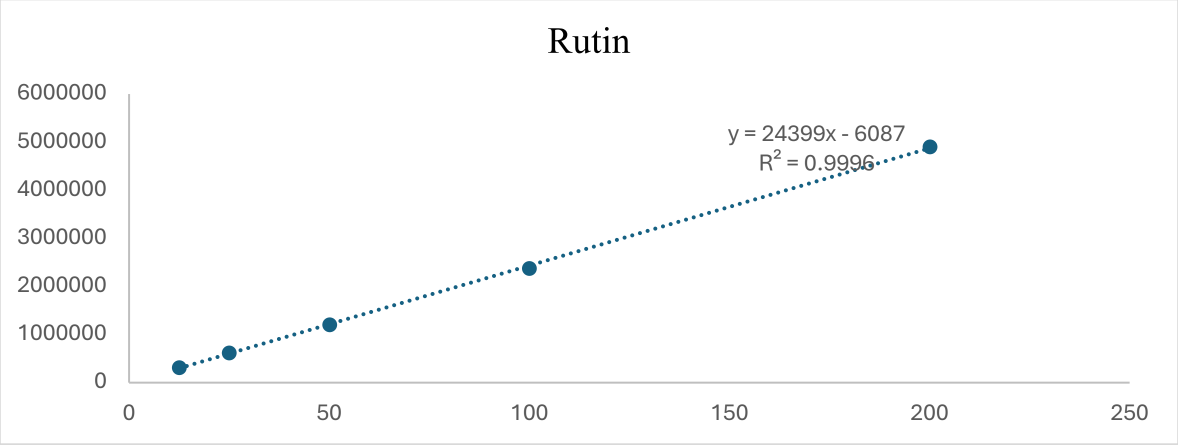

Quantitative analysis							
Sample	A1	A2	A3	Average A	µg rutin /2 mg EAOr		mg rutin/g EAOr
EAOr	56270	54824	54561	55218.33333	2.51261664	1256.30832	1.25630832

We certify that the following article

An Organic Fraction of *Oenothera rosea* L'Her Ex. Aiton Prevents Neuroinflammation in Rat Ischemic Model.

Alejandro Costet Mejía, Gabriela Trejo-Tapia *, Itzel Isaura Baca-Ibarra, Aida Araceli Rodríguez-Hernandez, Julio García-Hernández, Brenda H. Camacho-Díaz, Alejandro Zamilpa *

has undergone English language editing by MDPI. The text has been checked for correct use of grammar and common technical terms, and edited to a level suitable for reporting research in a scholarly journal.
MDPI uses experienced, native English speaking editors. Full details of the editing service can be found at
► https://www.mdpi.com/authors/english.

MDPI Multidisciplinary Digital Publishing Institute

Basel, Switzerland
August 2024

AUTHORservices

english-84449
